# Developing a faculty simulation-based education strategy: a Delphi study to build consensus and aid decision making

**DOI:** 10.1186/s41077-025-00393-9

**Published:** 2025-12-09

**Authors:** Nebras Alghanaim, Samantha Rogers, Jo Hart, Gabrielle Finn

**Affiliations:** 1 Division of Medical Education, Faculty of Biology, Medicine and Health, University of Manchester, Manchester, UK; 2 Faculty of Medicine, King Saud Bin Abdulaziz University for Health Sciences, Jeddah, Saudi Arabia; 3 King Abdullah International Medical Research Center, Jeddah, Saudi Arabia; 4 Ministry of the National Guard - Health Affairs, Jeddah, Saudi Arabia; 5 Division of Nursing, Midwifery & Social Work, Faculty of Biology, Medicine and Health, University of Manchester, Manchester, UK

## Abstract

**Background:**

Simulation-based education (SBE) improves learner competence, patient safety and workforce readiness. Yet, existing frameworks such as INACSL and ASPiH provide limited guidance on developing sustainable, institution-wide strategies, particularly in decentralised, multidisciplinary contexts. This study aimed to create a faculty-wide SBE strategy using the e-Delphi method to build consensus among diverse stakeholders.

**Method:**

The study was conducted at a UK higher education institution without a centralised simulation centre. An e-Delphi process was used to refine strategic statements across three survey rounds. Panellists were purposively sampled and included internal and external academics, postgraduate and undergraduate students, and international contributors from 7 countries and 15 healthcare-related professions. Initial statements were derived from established SBE frameworks and refined based on quantitative agreement (≥ 80% consensus) and thematic analysis of free-text feedback.

**Result:**

Of 111 invited participants, 41 completed Round 1, increasing to 43 in Rounds 2 and 3. The process produced 39 final strategic statements grouped under eight strategic priorities: (1) Leadership and governance; (2) Communication and networking; (3) Training and development; (4) Standards and quality assurance; (5) Research and evaluation; (6) Accessibility; (7) Preparation and planning; and (8) Finance. These priorities map to three overarching themes—Connectivity, collaboration and partnership; Promoting quality; and Stability, sustainability and growth of SBE. The strategy embeds SBE into institutional processes, aligning it with budgeting, infrastructure planning, workforce development, and digital transformation. It emphasises multi-level governance, sustainability planning, technology integration and inclusivity through student patient and public involvement and engagement (PPIE) representation.

**Conclusion:**

The e-Delphi method effectively built consensus on a comprehensive SBE strategy tailored to a decentralised, multidisciplinary faculty. The strategy goes beyond existing frameworks by integrating sustainability, multi-level governance, and structured technology planning, while embedding student and PPIE perspectives. It offers a scalable, replicable model for institutions seeking to align simulation provision with strategic priorities, accreditation standards and equitable access. Future research should examine the strategy’s impact on educational outcomes, workforce readiness and its adaptability across disciplines and institutional contexts.

## Introduction

Simulation-based education (SBE) has become an integral component of healthcare curricula, driven by growing evidence of its impact on learner competence, patient safety and clinical preparedness. Not all higher education institutions (HEIs) have centralised simulation centres with established governance structures. In decentralised simulation centres or developing environments, SBE often evolves in a fragmented manner, without a cohesive strategy or central oversight [1, 2]. This challenge is particularly pronounced in multidisciplinary faculties, where simulation provision may be diverse but uncoordinated, limiting sustainability, equitable access and strategic alignment. This study addresses this gap by exploring an inclusive, consensus-driven approach to strategy development within such a context.

Despite the proliferation of SBE standards, HEIs continue to face significant challenges in developing sustainable, institution-wide strategies. Frameworks such as the INACSL standards of best practice [3–7], the Society for Simulation in Healthcare (SSH) accreditation standards [8] and the ASPiH Simulation Standards [9, 10] provide essential benchmarks for quality and consistency but offer limited guidance on strategic implementation within complex institutional structures. As a result, HEIs are often left to design their integration models independently, leading to fragmented efforts, duplicated resources and difficulties in achieving long-term sustainability [11, 12].

Across HEIs, SBE is delivered through a variety of structural models. Centralised simulation centres can offer significant advantages, including shared infrastructure, standardised processes and strategic oversight. However, they are not without limitations. Centrally governed models may fail to engage discipline-specific stakeholders or align with local pedagogical priorities, resulting in perceived disconnects between governance and practice [13]. Conversely, decentralised models, where simulation is embedded within individual programmes, may promote disciplinary relevance and responsiveness but can lead to inconsistencies in delivery, duplication of resources and limited interdisciplinary collaboration [14]. In such contexts, the absence of a unifying governance structure poses a significant barrier to embedding simulation strategically or aligning it with broader institutional goals [12].

At our institution, there is no centralised simulation centre. Instead, simulation is delivered across a large, multidisciplinary faculty that includes audiology, biosciences, medicine, nursing, midwifery, dentistry, pharmacy, optometry, psychology, public health and data, speech and language therapy and social work. Each programme operates independently, with its own simulation facilities, programme-specific goals, accreditation standards and clinical training models. While this diversity reflects the breadth and complexity of modern healthcare education, it also results in limited resource sharing, few opportunities for interdisciplinary collaboration and inconsistent simulation practices across departments. The decentralised nature of our simulation provision highlighted the urgent need for a coherent, faculty-wide strategy to reduce duplication, support equity of access and align SBE with broader institutional goals. A structured SBE strategy was therefore essential for ensuring viability, effective resource allocation and staff engagement. Our institution needed to move beyond basic accreditation compliance and instead invest in an integrated approach that supports cost-effectiveness, educational quality and sustainable infrastructure.

Although some institutions have reported on local simulation initiatives or centre development, there remains limited research detailing how HEIs have developed and implemented faculty-wide SBE strategies, particularly in decentralised, multidisciplinary environments. Most available publications focus on simulation programme design, accreditation processes or operational standards, rather than on the governance, leadership and planning mechanisms necessary for institutional coherence [15, 16]. While existing frameworks imply the importance of elements such as faculty development, quality assurance and technological integration, few studies explore how these components are prioritised and operationalised across complex academic structures [17].

Given these challenges, there is a clear need for structured, inclusive methods that enable institutions to develop coherent, faculty-wide SBE strategies. Traditional consensus-building approaches, such as focus groups, in-person workshops or nominal group technique, may be limited by logistical constraints, power dynamics or lack of anonymity [18, 19]. The Delphi method offers a robust alternative, supporting anonymous participation, structured iteration and the synthesis of diverse expert perspectives [20, 21]. In large, multidisciplinary faculties, the e-Delphi format further enhances accessibility and inclusivity by accommodating participants across geographic, disciplinary and professional boundaries [22].

This paper reports on the use of a structured Delphi process to develop a consensus-driven simulation strategy within a large, decentralised faculty. By engaging a diverse panel of academic, clinical and student stakeholders in an inclusive, iterative process, the study generated a strategic framework that reflects shared priorities across disciplines and roles, supporting relevance, sustainability and institutional alignment. This work was undertaken as part of a broader strategic initiative to develop both a faculty-wide SBE strategy and a complementary interprofessional education (IPE) strategy [23], reflecting the institution’s commitment to integrated and future-facing healthcare education. This paper focuses specifically on the SBE component. The approach offers a scalable model for other HEIs seeking to strengthen simulation governance, promote equity of access and enhance interdisciplinary collaboration.

### Aim of the study

This study aimed to develop a faculty-wide simulation-based education (SBE) strategy through consensus-building with diverse stakeholders using the Delphi method.

## Methods

### Research question

What are the key components of a faculty-wide SBE strategy developed through a Delphi consensus process?

### Study design

The E-Delphi method, an evolution of the traditional Delphi technique developed by Dalkey and Helmer [24], is widely adopted in health and educational research to enhance decision-making by achieving consensus on healthcare standards and strategic initiatives [22, 25, 26]. E-Delphi offers a more cost-effective and efficient alternative, allowing participants to contribute from any location and at any time [20]. E-Delphi follows the Delphi principles of anonymity, controlled feedback, iterative rounds and statistical aggregation, ensuring consensus while minimising social pressures [21]. The study adhered to the DELPHISTAR guidelines for reporting [27], reinforcing transparency, credibility and reproducibility in the Delphi process. By using at least two iterative rounds, anonymity and equal participant status is maintained, preventing dominant voices from influencing outcomes [19, 28, 29]. Ultimately, the E-Delphi method enhances the validity and quality of consensus-driven research, contributing to best practices in healthcare and education.

### The study setting

The study took place at a single UK higher education institution within the Faculty of Biology, Medicine and Health. The Delphi process was embedded within this faculty, which encompasses 13 academic programmes across a range of healthcare disciplines. Each programme delivers SBE independently, with no central governance or unified infrastructure. These formed the rationale for developing a faculty-wide SBE strategy to support greater cohesion, equity, and sustainability.

### Establishing the research team

This research was undertaken by an interdisciplinary team from medical education, nursing and psychology departments, with expertise in healthcare education and Delphi methods.

### The Delphi process

#### Developing the initial strategic statements from best practice

The research team created a list of strategic statements for the expert panel, ensuring that key goals and priorities aligned with best SBE practices. To achieve this, the team conducted a comprehensive review of existing SBE frameworks, competencies and standards, developing 24 strategic statements. This review included key publications such as INACSL [3–7], SSH [8], ASPiH [9], ASPiH 2023 [10] and the National Framework for Simulation-Based Education [30].

To systematically analyse these frameworks, the research team outlined the criteria and standards from each framework in an Excel spreadsheet, highlighting commonalities across key operational areas. The focus was on critical components of SBE, including leadership and governance, quality assurance, financial planning, infrastructure and sustainability. These elements were then aligned with the institution’s broader strategic goals, ensuring that the SBE strategy adhered to established best practices and supported long-term institutional priorities. This structured approach facilitated developing a cohesive, scalable and institutionally relevant SBE strategy.

The strategic statements were organised under three main priorities, each accompanied by strategic objectives as shown in Table 1.

**Table 1:** Strategy structure

Strategic priority	Strategic objectives
**1. Connectivity, collaboration and partnership**	a. Leadership and governance b. Communication and networking
**2. Promoting quality**	a. Training and development b. Standards and quality assurance c. Research and evaluation
**3. Stability, sustainability and growth of simulation-based education**	a. Accessibility b. Preparation and planning c. Finance

### Establishing the Delphi panel

#### Selecting the Delphi panel

The panel was comprised of individuals with significant knowledge or skills related to the study’s focus [20]. Participants were required to have sufficient expertise in delivering SBE to undergraduate or postgraduate healthcare students or participating in SBE. Panellists were identified through purposive sampling, utilising a ‘snowballing’ technique to ensure sufficient diversity [31] of various disciplines across healthcare programs.

Educational leaders from schools involved in SBE, such as programme leads, were asked to send information about the study to potential participants considered knowledgeable in SBE. The principal researcher also directly contacted international academics to broaden representation. Involving international panellists was a deliberate decision to enrich the strategy with globally informed insights. Drawing on diverse expertise allowed the research team to incorporate best practices, experiences and innovations from different health systems and educational contexts. This global perspective helped foster adaptability, interprofessional collaboration [32] and cultural sensitivity, qualities increasingly essential for academic institutions operating in an interconnected and dynamic healthcare landscape. Although the strategy was developed for a single institution, these external viewpoints ensured its design was forward-thinking, evidence-informed and aligned with international trends, ultimately enhancing its credibility and transferability across similar settings [16].

The principal researcher also contacted undergraduate and postgraduate students from inside and outside the university. Both groups were enrolled as co-developers of the strategy [33, 34]. Their participation ensured that the strategy remained relevant and inclusive by addressing their needs, promoting a sense of ownership and enabling them to engage in decision-making. This co-development approach provided feedback, spurred innovation and built trust between students and the institution. Strategies developed with co-developers' input are more likely to succeed and achieve widespread acceptance, ensuring sustainability while encouraging growth and a stronger sense of community [33, 34]. The eligibility criteria are shown in Table 2.

**Table 2:** Panel eligibility criteria

Group	Eligibility criteria
**Academics and clinical practitioners:**	• Had at least two years of experience teaching in SBE • Demonstrated involvement in research related to SBE • Held a degree or postgraduate qualification specialising in teaching and learning
**Postgraduate and undergraduate students:**	• Had experience in SBE as a learner or an educator • Completed research in SBE, such as a master’s thesis or PhD dissertation

#### Panel size

Although there is no universally accepted ideal panel size for the Delphi process [22], a sample size of 15 to 30 participants is usually regarded as sufficient for most studies [20, 35], or a larger heterogeneous panel with a range of 5 to 10 in each area of the group, to maintain its representativeness and the value of the group [26].

#### Recruitment

The primary researcher was responsible for recruiting and contacting potential panel members, who had been previously identified and selected by the research team according to the criteria outlined in Table 2. The invitation email included a study overview, estimated timelines for each Delphi round, a participant information sheet and a consent form, which was obtained before the first round from those who agreed to participate.

### Data collection

The E-Delphi survey was conducted over three rounds between September and December 2023, as previously defined to the panel in the invitation email. The E-surveys were designed using Qualtrics software [36], an online survey data collection platform. Survey links were emailed to all participants throughout the Delphi rounds [18]. Participants were given approximately three weeks to complete each round and weekly reminders were sent to maintain the stability and performance of the panel contribution [26]. This structured approach ensured a thorough and collaborative refinement process for the strategic statements. A detailed data collection approach for each round is presented in Table 3.

**Table 3:** Data collection approach in each round

Round	Round approach
**Round 1**	All participating panellists reviewed the research team's initial statements and were requested to accept, reject or modify them and propose new strategic statements
**Round 2**	Participants reviewed the revised statements according to Round 1 findings. In Round 2, participants were asked to further accept, reject or modify the revised statements
**Round 3**	Panellists were asked to accept or reject each strategic statement definitively following Round 2 results

We implemented the accept, reject, modify method due to its benefits over the Likert Scale with Delphi findings, especially for reaching an actionable consensus [19, 37]. This approach necessitates clear decisions, making data analysis easier and reducing uncertainty and bias [19, 37], and simplifies interpretation and allows for more efficient refinement of items across rounds, which allows the panel to propose changes, facilitates the ongoing enhancement of contexts or statements [19, 37]. This method emphasises practical results and organised decision-making, effectively collecting feedback [19, 37]. During all three rounds, participants were encouraged to share qualitative feedback via a free-text comment box. These narrative responses enriched the data by offering deeper insights, justifications and suggestions for modifying the statements, thereby supporting a more nuanced understanding of panel perspectives.

Consensus was operationalised using a predetermined threshold of ≥ 80% agreement among participants. Statements receiving ≥ 80% agreement to accept were retained and carried forward, often with revisions based on feedback. Statements that received ≥ 80% agreement to reject were removed from subsequent rounds. Items with mixed responses were revised and re-presented depending on the content and clarity of panel feedback. Items with less than 80% consensus were excluded. This iterative process allowed for both quantitative consensus and qualitative refinement, ensuring the final set of statements was both representative and practically applicable.

### Data analysis

The panel’s engagement was pivotal to the success of the E-Delphi study, which aimed for a response rate of over 70% to ensure result validity and reliability [4]. After each round, the research team analysed levels of agreement, with the consensus threshold pre-set at 80% agreement [3, 35, 38]. Statements failing to meet this threshold were excluded in subsequent rounds.

Following each round, qualitative feedback from participants, collected via a free-text comments box, was analysed using thematic analysis [31] and common themes and points of divergence were extracted. Thematic analysis is a method for examining qualitative data through the identification and interpretation of recurring patterns [39]. It enables researchers to capture diverse participant perspectives, highlight similarities and differences and reveal unexpected insights [40]. The process typically involves systematic coding to ensure consistency and reduce researcher bias [41, 42]. This study followed Braun and Clarke’s six-phase approach [39, 43]: familiarisation, coding, theme generation, reviewing, refining and defining themes and reporting. The manual analysis was done independently and then reviewed by the team. This informed the creation or revision of statements subsequently presented to participants for further rating or discussion. Statements that achieved consensus yet contained uncertain or vague comments lacking sufficient detail for development were revisited in later rounds. In a similar format, these statements were re-evaluated to gather additional input and clarification from the panel, ensuring they were refined and aligned with the study aim. To promote consistent decision-making, the research team documented the impact of qualitative feedback on subsequent iterations. This approach enhanced transparency [19] and established an audit trail.

### Ethical approval

This study had ethical approval from the University of Manchester (UoM) Proportionate University Research Ethics Committee (UREC) (Reference 2023–16610–30096). It was conducted in accordance with institutional ethical standards, the Declaration of Helsinki and the Health Research Authority where applicable. Participation was entirely voluntary, with the option to withdraw at any time. No incentives were provided.

## Results

### Overview of the panel

Out of 111 participants invited to contribute to the study, 41 participated in Round 1, with participation increasing to 43 in Rounds 2 and 3. Round 1 achieved a response rate of 85.4% (*n* = 35), followed by 67.4% (*n* = 29) in Round 2 and 62.7% (*n* = 27) in Round 3.

The panel comprised of internal and external academics, postgraduates and a small number of undergraduates, offering perspectives from across disciplines and levels of study. The panel consisted of participants from 7 countries and 15 health-care related professions, providing an invaluable international and interprofessional/disciplinary perspective to the study. Audiology, dentistry, psychology, social work and public health and data were not represented. Countries and professions of the Delphi Participants are illustrated in Table 4:

**Table 4:** Countries and professions of the Delphi participant across all three rounds

Countries and professions of the Delphi participants	
**Countries**	Number and percentage
UK	(23) 55.8%
Saudi Arabia	(10) 25.5%
USA	(3) 6.9%
Canada	(2) 4.6%
Australia	(2) 4.6%
New Zealand	(2) 4.6%
Portugal	(1) 2.3%
**Professions**	Number and percentage
Medical doctor	(11) 25.5%
Registered nurse	(9) 20.9%
Pharmacist	(7) 16.2%
Paramedic	(3) 6.9%
Speech and language therapist	(2) 4.6%
Optometrist	(2) 4.6%
Occupational therapist	(1) 2.3%
Respiratory therapist	(1) 2.3%
Midwife	(1) 2.3%
Radiation therapist	(1) 2.3%
Dietitian	(1) 2.3%
Biomedical engineer	(1) 2.3%
Chemist	(1) 2.3%
Biologist	(1) 2.3%
Biological anthropologist	(1) 2.3%

#### Overview of strategy development

**Fig. 1:**
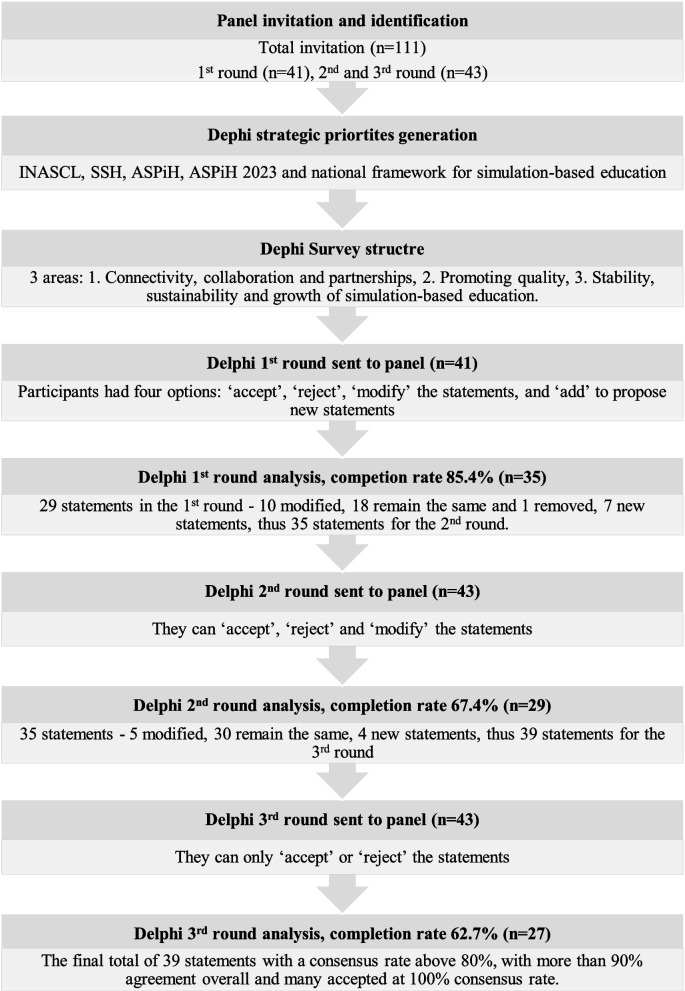
Delphi study overview flowchart

Figure 1 presents a comprehensive summary of the Delphi study process, panel identification and completion rates, survey structure and the strategic development of each round.

#### Survey completion rates and demographic composition by round

Survey completion rates were as follows: Round 1 (*n* = 35; 85.4%), Round 2 (*n* = 29; 67.4%), and Round 3 (*n* = 27; 62.7%). The demographic composition of a panel at each round is shown in Table 5.

**Table 5:** Survey completion rates and demographic composition by round

Round	Survey completion rates and demographic composition by round
**Round 1**	Round 1 (*n* = 35; 85.4%): Internal academics (*n* = 11), external academics (*n* = 13), postgraduate (*n* = 8) and undergraduate students (*n* = 3)
**Round 2**	Round 2 (*n* = 29; 67.4%): Internal academics (*n* = 9), external academics (*n* = 9), postgraduate (*n* = 8) and undergraduate students (*n* = 3)
**Round 3**	Round 3 (*n* = 27; 62.7%): Internal academics (*n* = 8), external academics (*n* = 9), postgraduate (*n* = 7) and undergraduate students (*n* = 3)

#### Strategic statement creation

At the start of the study, 29 strategic statements were distributed across the three priority areas. At the end of the E-Delphi process, the number of statements had increased to 39, as shown in Table 6.

**Table 6:** Strategic statement creation in each round

Round	Strategic statement creation in each round
**Round 1**	Out of 29 initial statements, 18 were accepted, 10 were revised and re-included in Round 2, 1 was removed due to low consensus and 7 new statements were added
**Round 2**	Of 35 statements (the revised 10 from Round 1 plus 7 additions), 30 were accepted, 5 were revised and re-included in Round 3 and 4 new statements were added
**Round 3**	All 39 statements were accepted as outlined in Table 9

Participants’ qualitative feedback via free-text comments highlighted the need for clear leadership definitions, sustainable infrastructure systems, maintenance, faculty development frameworks, robust evaluation methods and expanded simulation integration across disciplines. Table 7 summarises the comments/feedback. Table 8 presents an example of the Delphi analysis process, illustrating how a statement was evaluated and refined.

**Table 7:** Summary of qualitative comments/feedback

Theme	Delphi Panel qualitative comments/feedback
**Leadership**	• Define terminology, e.g. ‘community of practice’, ‘academic lead’, ‘instructor’, ‘simulation coordinator’, ‘digital platforms’, ‘infrastructure’, ‘impressive technology’ and newly qualified registrants • Leadership should be clearly defined • Appoint student representatives for input in SBE development
**Sustainable infrastructure systems and facilities**	• Develop a structured system for equipment maintenance and auditing for sustainability • Issues or concerns about staffing, staff employment, supporting or development • Address integration of VR, AI and game-based simulation technologies
**Faculty development and perspectives**	• Develop a continuous faculty development framework • Ensure ongoing professional development in SBE
**Evaluation**	• Establish a periodic and multi-stakeholder evaluation framework • Define valid and reliable assessment tools for faculty competency evaluation
**Future integration**	• Expand simulation beyond healthcare to other faculties (e.g., humanities using VR) • Ensure integration of undergraduate and postgraduate simulation experiences

**Table 8:** Example of Delphi analysis and on-process on a statement

	Initial statement and results	Comments from 1^st^ round (category of comment)	2^nd^ round statement and results	Comments from 2^nd^ round	3^rd^ round statement – final and result
2. Promoting quality 2.3 Research and evaluation	2.3.3 Continue to encourage and support dissemination of outcomes/findings from research and evaluation processes in professional journals, internal and external conferences Result: Accept: (31) 88.75% Reject (0) 0% Modify (4) 11.43%	* There must be a way to make this more meaningful (Modification) *Encourage and support dissemination of outcomes/findings from research and evaluation processes in professional journals, scientific publications, dissemination in regional/national/international conferences (Modification) *Should research and evaluation is not separate. We should aim to publish research but not the routine evaluation (Separate statement) *Research requires funding and supervision. There is no clear process for funding for pedagogical research (Funding) *Employ research lead to help guide academics delivering simulation who may want to do some form of research (Staffing) *Disseminate the evaluation results internally (with proper anonymisation), promoting recognition and improvement at an individual, division, school and faculty level (New statement suggestion)	2.3.3 Continue to encourage and support dissemination of outcomes/findings from research and or evaluation processes in professional/scientific journals, internal and external conferences Result: Accept (27) 93.10% Reject (0) 0% Modify (2) 6.90%	*Remove 'continue to encourage' and replace with something more concrete around setting up systems to actively support and promote this	2.3.3 Establish systems to actively support and promote the dissemination of outcomes/findings from research and/or evaluation processes in professional/scientific journals, and internal and external conferences Result: Accept (27) 100% Reject (0) 0%
			2. 3.4 Disseminate evaluation data internally (with proper anonymisation), promoting recognition and improvement at an individual, division, school, and faculty level Result: Accept (27) 93.10% Reject (0) 0% Modify (2) 6.90%	None	2.3.4 Disseminate evaluation data internally (with proper anonymisation), promoting recognition and improvement at an individual, division, school, and faculty level Result: Accept (27) 100% Reject (0) 0%

#### Key components of the SBE strategy

Consensus agreement ranged from 80 to 100% across rounds. The final 39 strategic statements were grouped under eight strategic priorities, each linked to one of the three overarching themes: (1) Connectivity, collaboration and partnership; (2) Promoting quality; and (3) Stability, sustainability and growth of SBE.

1. Leadership and governance
  Designated simulation leads with defined academic and technical roles.
  Multi-level governance structures spanning academic, technical, administrative and executive domains.
  Clear role definitions and accountability mechanisms to ensure institutional alignment.
2. Communication and networking
  Creation of faculty-wide communities of practice and steering groups to share expertise and best practices.
  Development of digital platforms to facilitate communication, collaboration and resource sharing.
  Regular engagement with external stakeholders, professional bodies and regulatory organisations.
3. Training and development
  Equitable access to staff development in simulation pedagogy and emerging technologies.
  Structured professional development pathways for academic, technical and simulated patient staff.
  Internal mentorship and peer-shadowing opportunities to sustain skills and expertise.
4. Standards and quality assurance
  Embedding recognised simulation standards (INACSL, ASPiH, SESAM) into all activities.
  Regular quality review cycles, incorporating stakeholder feedback and programme evaluation.
  Peer-review and training needs analysis to maintain high-quality delivery.
5. Research and evaluation
  Ongoing evaluation of simulation’s impact on learning outcomes, patient safety and workforce readiness.
  Support and training for staff to develop and lead research in simulation-based education.
  Systems to actively disseminate research and evaluation findings internally and externally.
6. Accessibility
  Equitable access to simulation spaces, equipment and opportunities across all programmes.
  Representation of diverse patient populations in manikins, scenarios and simulated patients.
  Faculty-wide systems for equipment and facility sharing to optimise use.
7. Preparation and planning
  Forecasting future simulation needs, including staffing, infrastructure and technology.
  Use of readiness assessment tools to support strategic growth and integration of simulation.
  Quality assurance frameworks to guide expansion into new programmes.
8. Finance
  Operational and capital budget planning aligned with strategic priorities.
  Long-term financial models to support sustainability and scalability.
  Resource allocation processes that maximise efficiency and value.

These eight priorities form a comprehensive, consensus-driven framework designed to embed SBE within the institution’s strategic vision while ensuring sustainability, inclusivity and innovation. The detailed statements for each priority are provided in Table 9.

**Table 9:** The final SBE strategies

**Strategic priority (1): Connectivity, collaboration and partnerships**	
**Aim:**	To establish a shared vision for simulation based on our identity as a learning community within the faculty
**We will address this through:**	i. Leadership and governance: Establishing faculty infrastructure that supports the goals and outcomes of simulation
	ii. Communication and networking: Maximising communication and networking opportunities to share best practices and facilitate connectivity, collaboration and partnership between simulation academic staff/faculty members, learners, patients, service users and external stakeholders
**i. Leadership and governance**	
1. Appoint a lead/s for simulation to lead the development and implementation of the simulation strategy and report progress to the faculty leadership team. Leadership will be clearly defined and appropriate governance models and processes will be explicitly described	
2. Review and clarify academic programme and technical support structures and leadership roles in relation to simulation and articulate roles and responsibilities to ensure parity across faculty (including workload tariff) and goals of simulation regularly (e.g., every two years)	
3. Develop and facilitate collaborative working relationships with technical services operational managers to better understand the roles and responsibilities of simulation technicians/technologists and ensure colleagues have clear career pathways with access to ongoing training and development	
4. Develop and facilitate collaborative working relationships with information technology (IT) Services and E-Learning support teams to promote the sharing of ideas, taking responsibility for innovation and best practices in using simulation and immersive technologies to enhance the learning experience	
5. Develop and facilitate ongoing relationships with executive stakeholders, faculty/organisational development teams, quality improvement and assurance, teaching and learning teams	
6. Appoint student representatives with clearly defined roles and responsibilities to inform the development of simulation	
7. Work with the social responsibility and public engagement team to ensure strategies, plans and goals align with patient and public involvement and Engagement (PPIE) principles, e.g., PPIE representation in steering groups	
**ii. Communications and networking**	
8. Establish a community of practice and/or steering committee with clear mechanisms to share best practices, learning and expertise across all university healthcare programmes, including cooperation with, for example, but not limited to, the Association for Simulated Practice in Healthcare (ASPiH), International Nursing Association for Clinical Simulation and Simulation Learning (INACSL), Society for Simulation in Europe (SESAM) and Association of Standardized Patient Educators (ASPE)	
9. Develop a digital platform/virtual learning environment to promote effective communication pathways, share resources (e.g. iRIS and e-learning for health) and expertise, showcase best practices and facilitate collaborations across simulations within the university	
10. Continue to develop and establish liaisons with external stakeholders relevant to individual healthcare simulation training requirements, including professional regulatory and statutory bodies, Royal Colleges and NHS (National Health Service) Trusts, ensuring protected time for discussion via regular meetings	
**Strategic priority (2): Promoting quality**	
**Aim:**	To continue delivering high-quality simulation that promotes learners’ ability to meet identified learning outcomes/objectives
**We will address this through:**	i. Training and development: Providing equitable access to simulation staff development programmes
	ii. Standards and quality assurance: Promoting quality-assured simulation and staff training across the professional spectrum
	iii. Research and evaluation: Fostering evaluation processes that promote a reflective learning culture leading to improvement
**i. Training and development**	
11. Provide new and existing academic staff/faculty members delivering simulation with flexible and accessible training opportunities in simulation pedagogy as part of continuing professional development by completion of simulation development programmes such as, but not limited to, the Certified Healthcare Simulation Educator (CHSE), and Simulation Faculty Development Programme (e.g., e-lfh.org.uk)	
12. Provide new and existing academic staff/faculty members delivering simulation with flexible and accessible training opportunities in immersive technology, e.g., virtual reality, artificial intelligence and serious gaming based on the curriculum and intended learning outcomes for programmes	
13. Support new and existing academic staff/faculty members by delivering simulations to continue developing knowledge and skills in the debriefing process, including meta-debriefing as appropriate	
14. Support academic staff/faculty members delivering simulation to participate in advisory committees, professional or practice-based simulation forums or networks as part of continuing professional development	
15. Develop and implement a roadmap for professional development designed specifically for academic staff/faculty members delivering simulation. The professional development plan and/or pathway should include, but not be limited to, membership and engagement with professional simulation networks, attendance at local/regional/national/international conferences, completion of simulated-based education study days/courses and achievement of individual accreditation with a relevant simulation association	
16. Support simulation technicians/technologists in the development of knowledge, skills and behaviours that will enable them to continue to provide consistent, high-quality simulation in safe learning environments by completion of professional registration with the Science Council, e.g., simulation technician level 3, and certified healthcare simulation operations specialist certification (CHSOS) scope	
17. Develop and implement an internal mentorship programme and/or peer-shadowing opportunities to provide continuous support and professional development of academic staff/faculty members/simulation technicians delivering simulation	
**ii. Standards and quality assurance**	
18. Raise awareness and promote the application of healthcare simulation standards of best practice, including, but not limited to: Association for Simulated Practice in Healthcare (ASPiH), International Nursing Association for Clinical Simulation and Learning (INACSL), Society for Simulation in Europe (SESAM), Association of Standardized Patient Educators (ASPE) and Simulated Patient Common Framework Checklist (Health Education Northwest)	
19. Embed healthcare simulation standards of best practice into the design and development of all simulation activities and consider programme/organisational accreditation, as appropriate, with a relevant simulation association	
20. Ensure that staff designing and delivering simulations are knowledgeable of the ethical standards of simulation-based experiences and adhere to the Healthcare Simulationist code of ethics	
21. Use a periodic review and feedback process to ensure all simulation activities delivered across faculty are feasible, appropriately designed based on programmatic resources and in alignment with the simulation strategy. This will be measured by quality assurance processes, e.g., annual evaluation of programme simulation activities, incorporating outcomes data and learner, academic staff/faculty member and external stakeholders’ feedback	
22. Undertake a training needs analysis to identify training and development needs for academic staff/faculty members delivering simulation and simulation technologists/technicians, using, for example, the simulation educational needs assessment (SENAT) tool	
23. Engage in annual peer-review processes to ensure ongoing development of academic staff/faculty members delivering simulation	
24. Establish a clear process and/or system of reviewing simulation resources, e.g., standards of best practice, e-learning materials, evidence-based practice and training and development courses, to ensure academic staff/faculty member/simulation technologist/technicians remain up to date	
**iii. Research and evaluation**	
25. Commit to undertaking evaluations of all aspects of simulation activity (i.e., briefing or pre-brief, simulation activity, debriefing, simulated patient’s skills in portraying their role) to determine the quality and/or effectiveness of the simulation-based experience on an individual, divisional, school or faculty level. Evaluation should map to learning evaluation models, e.g., Kirkpatrick, and include feedback from learners, academic staff/faculty members, simulated/standardised patients, equality, diversity and inclusion (EDI) leads and external stakeholders	
26. Facilitate appropriate training and supervision for academic staff/faculty members designing and delivering simulations to develop research projects and evaluation processes that consider educational effectiveness and efficiency, patient safety, quality of care and the preparedness of learners for the workforce	
27. Establish systems to actively support and promote the dissemination of outcomes/findings from research and/or evaluation processes in professional/scientific journals, and internal and external conferences	
28. Disseminate evaluation data internally (with proper anonymisation), promoting recognition and improvement at an individual, division, school and faculty level	
**Strategic priority (3): Stability, sustainability and growth of simulation-based education**	
**Aim:**	Embrace current and future developments in simulation to enhance the learner's experience
**We will address this through:**	i. Accessibility: Improving accessibility, deliverability and utilisation of simulation teaching facilities and equipment; striving for equitable access across healthcare programmes
	ii. Preparation and planning: Identifying priority areas for current and future developments
	iii. Finance: Securing and managing the financial resources to support stability, sustainability and growth of simulation delivery
**i. Accessibility**	
29. Review current specialist teaching spaces with a view to developing a system/process for sharing spaces, e.g., aseptic suite, to increase capacity for simulation delivery and enhance learner’s experience of simulation	
30. Map existing simulation equipment and auditing processes, e.g., part-task trainers, full-body manikins, advanced procedural trainers and VR (virtual reality) headsets, with a view to developing a system/process for sharing equipment to increase capacity for simulation delivery	
31. Ensure full-body manikins, part-task trainers and avatar-based simulation represent all patient populations, e.g., race, ethnicity, age, various body sizes and disability, to promote equity, diversity and inclusion	
32. Review the use and training of simulated patients across the faculty, with a view to establishing a pool of simulated patients, ensuring that they are trained for the roles that they are required to undertake, including providing feedback and debriefing in line with evidence-based practice and reflect all patient populations to promote equity, diversity and inclusion	
33. Identify a learning space to build and develop an innovative simulation centre/hub to increase capacity for simulation delivery, including interprofessional-enhanced simulation	
34. Ensure digital innovations are accessible for all learners, ensuring an inclusive approach to teaching and learning	
**ii. Preparation and planning**	
35. Assess academic staff/faculty member readiness for simulation growth, e.g., workload, role and responsibility, training and development needs	
36. Forecast programme/faculty growth for simulation, including personnel (academic staff/faculty member, simulation technicians/technologists), Information technology (IT), E-learning, librarian support, workload, roles and responsibilities, training and development needs, simulation equipment and facilitates, ensuring equity of access for learners across all healthcare programmes	
37. Explore and identify priorities, benefits, challenges and solutions for incorporating simulation and immersive technologies into all healthcare programmes within the faculty, using, for example, the Simulation Culture Organizational Readiness Survey (SCORS)	
38. Develop and implement a quality assurance framework to enable continuous progress in simulation preparation, planning, delivery and integration into new healthcare programmes	
**iii. Finance**	
39. Prepare an operational budget considering current and future goals and priorities, including identifying fixed (e.g., maintenance and service contracts), variable (e.g., personnel, reimbursements for simulated patients, consumable items, training and development for staff and simulated patients, peer review, audit, dissemination of research and scholarly activity) costs, future capital expenditure, and human resources	

## Discussion

This study aimed to identify the key components of a faculty-wide simulation-based education (SBE) strategy through a structured Delphi consensus process. The strategy was developed for a decentralised faculty, where simulation is delivered independently across multiple programmes without a central simulation centre, addressing challenges in coherence, resource sharing and equity of access [1, 2].

Several features distinguish the strategy from existing SBE frameworks. It extends beyond programme-level educational focus, embedding simulation within institutional systems and integrating financial planning, infrastructure, IT and governance. While existing standards, such as INACSL [3–7] and ASPiH [10], prioritise faculty development and quality assurance, our approach takes a wider institutional view. This positions SBE as both a pedagogical enhancement and a catalyst for institutional excellence, student success and healthcare workforce preparedness [16]. The breadth of integration, spanning finance, workforce, infrastructure and digital strategy, may offer a transferable model for other institutions seeking to embed simulation within strategic planning.

A distinguishing feature is the structured leadership and governance model spanning academic, technical and administrative domains. While INACSL and ASPiH acknowledge governance, they often emphasise faculty training and operational oversight rather than multi-level governance. By clearly defining roles, establishing stakeholder engagement mechanisms and fostering interdisciplinary partnerships, our strategy embeds SBE as an institutional priority. This aligns with calls for distributed but coordinated leadership to enhance organisational resilience and cross-departmental engagement [44, 45]. Student representation ensures learner perspectives shape SBE development and accessibility; formal IT collaboration supports the integration of digital technologies; and patient and public involvement and engagement (PPIE) strengthens authenticity, equity and societal relevance.

The Delphi process itself offers a point of distinction. Previous SBE-related Delphi studies have often focused on defining competencies, prioritising curriculum elements or setting operational standards [21, 29]. Few have applied the method to develop a comprehensive faculty-wide strategy in a decentralised, multidisciplinary context. Our panel composition, combining internal and external academics, postgraduate and undergraduate students and international contributors, broadened the perspective and allowed the strategy to reflect both global trends and local realities.

Sustainability is central. Many SBE initiatives face funding volatility, resource constraints, and fragmented implementation, limiting long-term impact. Our strategy embeds sustainability planning within institutional frameworks, ensuring SBE is prioritised alongside organisational goals, workforce development, and healthcare needs. Whilst INACSL [3–7] and ASPiH [10] emphasise competency and operational standards, our approach explicitly addresses financial sustainability through long-term funding mechanisms, workforce capacity building via simulation integration in professional development pipelines and infrastructure maintenance to future-proof facilities. By linking SBE to budget planning cycles, we strengthen continuity, scalability and equitable access, avoiding budget shortfalls that can result in programme discontinuation [46].

Technological advancement is addressed as a strategic priority. The rapid growth of virtual reality (VR), artificial intelligence (AI) and game-based simulation necessitates institutional readiness and cross-disciplinary collaboration. While INACSL [3–7] and ASPiH [9, 10] recognise the importance of the technology, they offer limited guidance on institutional implementation or scaling. Our strategy provides a framework for integrating emerging technologies into curricula through cross-functional planning and partnerships between IT services, e-learning teams and simulation educators. It also includes scalable digital infrastructure, maintenance systems and shared platforms to ensure both longevity and equitable access. These measures align with evidence that immersive technology adoption must be planned strategically to achieve educational and operational sustainability [47].

The Delphi process also highlighted the value of embedding students and PPIE contributors in decision-making. Co-development has been shown to foster inclusivity, authenticity and stronger learner engagement [33, 34]. In our strategy, student representatives hold defined roles, contribute to steering groups and participate in evaluation processes. PPIE contributors bring patient and community perspectives into strategic discussions, promoting transparency, trust and responsiveness [48]. This participatory approach has been associated with improved authenticity in simulation design, better alignment with stakeholder needs and enhanced learner satisfaction [38, 49].

### Strength of the study

A key strength was the diversity of the panel, with participants from 7 countries and 15 professional disciplines, offering breadth of expertise that supports the strategy’s robustness and adaptability [26, 49]. This diversity enriched discussions on governance, sustainability and technology, and ensured the inclusion of multiple professional viewpoints. Involving international participants provided insights from a variety of healthcare and education systems, helping the strategy anticipate future developments and remain aligned with global trends [16].

Conducting the Delphi in a decentralised setting, without a central simulation centre, further increases its relevance for institutions with emerging or distributed simulation provision. The structured process offers a replicable model for aligning simulation efforts across disciplines through stakeholder engagement. While AI integration is often addressed at an institutional level, faculty engagement is essential to ensure its use is pedagogically appropriate and ethically sound [50].

### Limitations of the study

The panel was disproportionately composed of medicine and nursing participants, with relatively low numbers from disciplines such as audiology, social work and dentistry. While no evidence suggests that equal representation across professions improves outcomes and no maximum panel size has been defined [18, 26], some professions were underrepresented, potentially limiting the breadth of perspectives. This study brought together a large number of diverse panel from 7 countries and a broad spectrum of 15 disciplines from diverse professional backgrounds enriched the study, providing a comprehensive range of perspectives essential for developing a robust, equitable and inclusive education strategy which fosters reliability and dependability by including a large group of a wide range and a representative sample [26, 49]. This imbalance may have weighted judgements toward dominant professions and reduced discipline-specific nuance [18, 26]. Response rates declined in rounds 2 and 3, falling below the pre-specified 70% target. Reminder emails were issued to improve retention; nevertheless, attrition, typical in Delphi studies [51, 52], may have narrowed perspectives in later rounds. Non-responders were distributed across professions rather than clustered within a single discipline, reducing the risk of systematic bias influencing consensus outcomes [20]. Importantly, consensus thresholds (≥ 80%) were achieved, supporting the stability of the final statements despite lower participation. Finally, although conducted within a single institution, organisational differences (structure, culture, resources) may affect direct applicability elsewhere. However, contributions from 7 countries and 15 disciplines support transferability to comparable settings [18, 26].

### Implications and future considerations

The findings have implications for both institutional policy and external accreditation. By embedding recognised standards (INACSL, ASPiH) within a strategic framework, the strategy supports readiness for programme or organisational accreditation and facilitates benchmarking against national and international best practice. It also provides a blueprint for scaling SBE across other disciplines, including non-healthcare programmes where simulation is emerging as a pedagogical tool, such as social care, education and law.

The strategy offers practical guidance for curriculum designers, simulation leads and faculty developers and is under internal review for potential adoption across the faculty. Its integration into institutional processes supports both educational quality and sustainability. As SBE becomes increasingly central to preparing healthcare professionals for complex practice, further research should assess the academic and workforce impacts of institution-wide strategies across diverse contexts, including their adaptability to non-health disciplines.

## Conclusion

Through an e-Delphi process, we developed a consensus-driven SBE strategy tailored to a decentralised, multidisciplinary faculty. The strategy goes beyond existing frameworks by integrating sustainability, multi-level governance and technology planning, while embedding student and PPIE representation. It offers a scalable, replicable model for institutions seeking to align simulation with strategic priorities and accreditation standards. Future work should assess its impact on educational outcomes, workforce readiness and adaptability across disciplines and institutional structures.

## Data Availability

No datasets were generated or analysed during the current study.

## Abbreviations

SBE: Simulation-based education
INACSL: International Nursing Association for Clinical Simulation and Learning
SSH: Society for Simulation in Healthcare
ASPiH: Association for Simulated Practice in Healthcare
HEIs: Higher education institutions
UoM: University of Manchester

## References

[1] Okuda Y, Bryson EO, DeMaria S Jr, Jacobson L, Quinones J, Shen B, et al. The utility of simulation in medical education: what is the evidence? Mount Sinai J Med: J Trans Person Med. 2009;76(4):330–43. 10.1002/msj.20127. 10.1002/msj.20127 19642147

[2] Cheng A, Auerbach M, Hunt EA, Chang TP, Pusic M, Nadkarni V, et al. Designing and conducting simulation-based research. Pediatrics. 2014;133(6):1091–101. 10.1542/peds.2013-3267. 24819576 10.1542/peds.2013-3267

[3] Watts PI, McDermott DS, Alinier G, Charnetski M, Ludlow J, Horsley E, et al. Healthcare simulation standards of best practice simulation design. Clin Simul Nurs. 2021;58:14–21. 10.1016/j.ecns.2021.08.009.

[4] Rossler K, Molloy MA, Pastva AM, Brown M, Xavier N. Healthcare simulation standards of best practice simulation-enhanced interprofessional education. Clin Simul Nurs. 2021;58(2):49–53. 10.1016/j.ecns.2021.08.015.

[5] Miller C, Deckers C, Jones M, Wells-Beede E, McGee E. Healthcare simulation standards of best practice outcomes and objectives. Clin Simul Nurs. 2021;58:40–4. 10.1016/j.ecns.2021.08.013.

[6] McMahon E, Jimenez FA, Lawrence K, Victor J. Healthcare simulation standards of best practice evaluation of learning and performance. Clin Simul Nurs. 2021;58:54–6. 10.1016/j.ecns.2021.08.016.

[7] Charnetski M, Jarvill M. Healthcare simulation standards of best practice operations. Clin Simul Nurs. 2021;58:33–9. 10.1016/j.ecns.2021.08.012.

[8] Society for Simulation in Healthcare (SSH). Teaching and education accreditation (2021) standards revisions Society for Simulation in Healthcare (SSH). 2021. Available from: https://ssih.org/sites/default/files/2025-03/2021%20SSH%20Teaching-Education%20Accreditation%20Standards.pdf.

[9] Association for Simulated Practice in Healthcare (ASPiH). Simulation-based education in healthcare standards framework and guidance: Association for Simulated Practice in Healthcare (ASPiH). 2016:1–25. Available from: https://aspih.org.uk/wp-content/uploads/2017/07/standards-framework.pdf.

[10] Cristina Diaz-Navarro, Colette Laws-Chapman, Moneypenny M, Purva M. The ASPiH Standards – 2023: guiding simulation-based practice in health and care. Int J Healthcare Simul. 2024. 10.54531/nyvm5886.

[11] Boet S, Bould MD, Layat Burn C, Reeves S. Twelve tips for a successful interprofessional team-based high-fidelity simulation education session. Med Teach. 2014;36(10):853–7. 10.3109/0142159X.2014.923558. 25023765 10.3109/0142159X.2014.923558 PMC4245993

[12] Brown J. The seven silos of accountability in higher education: systematizing multiple logics and fields. J Am Coll Health. 2017;11:41–58.

[13] Kneebone R. Perspective: simulation and transformational change: the paradox of expertise. Acad Med. 2009;84(7):954–7. 10.1097/ACM.0b013e3181a843d6. 19550196 10.1097/ACM.0b013e3181a843d6

[14] Barth B, Arutiunian A, Micallef J, Sivanathan M, Wang Z, Chorney D, et al. From centralized to decentralized model of simulation-based education: Curricular integration of take-home simulators in nursing education. Cureus. 2022;14(6):e26373. 10.7759/cureus.26373. 35911310 10.7759/cureus.26373 PMC9329603

[15] Paul O’Connor, Emily O’Dowd, Lydon S, Byrne D. Developing a strategic plan for a healthcare simulation facility. Int J Healthcare Simul. 2022. 10.54531/gcih5434.

[16] Diaz-Navarro C, Armstrong R, Charnetski M, Freeman KJ, Koh S, Reedy G, et al. Global consensus statement on simulation-based practice in healthcare. Adv Simul. 2024;9:1–10. 10.1186/s41077-024-00288-1. 10.1186/s41077-024-00288-1 PMC11106913 38769577

[17] McGaghie WC, Issenberg SB, Petrusa ER, Scalese RJ. A critical review of simulation-based medical education research: 2003–2009. Med educ. 2010;44(1):50–63. 10.1111/j.1365-2923.2009.03547.x. 20078756 10.1111/j.1365-2923.2009.03547.x

[18] de Villiers MR, de Villiers PJ, Kent AP. The Delphi technique in health sciences education research. Med Teach. 2005;27(7):639–43. 10.1080/13611260500069947. 16332558 10.1080/13611260500069947

[19] Hsu CC, Sandford BA. The Delphi technique: making sense of consensus. Pract Assess Res Eval. 2007;12:10. 10.7275/pdz9-th90.

[20] Donohoe H, Stellefson M, Tennant B. Advantages and limitations of the e-Delphi technique. Am J Health Educ. 2012;43(1):38–46. 10.1080/19325037.2012.10599216.

[21] Varndell W, Fry M, Lutze M, Elliott D. Use of the Delphi method to generate guidance in emergency nursing practice: a systematic review. Int Emerg Nurs. 2021;56:100867. 10.1016/j.ienj.2020.100867. 32238322 10.1016/j.ienj.2020.100867

[22] Landeta J. Current validity of the Delphi method in social sciences. Technol Forecast Soc Change. 2006;73(5):467–82. 10.1016/j.techfore.2005.09.002.

[23] Alghanaim N, Rogers S, Finn G, Hart J. The creation of an interprofessional education (IPE) strategy utilising a delphi method. Clin Teach. 2025;22(3):e70098. 10.1111/tct.70098. 40320824 10.1111/tct.70098 PMC12050908

[24] Dalkey N, Helmer O. An experimental application of the Delphi method to the use of experts. Manag Sci. 1963;9(3):458–67. 10.1287/mnsc.9.3.458.

[25] Green RA. The delphi technique in educational research. SAGE Open. 2014;4(2):2158244014529773. 10.1177/2158244014529773.

[26] Landeta J, Lertxundi A. Quality indicators for Delphi studies. Futuers Foresight Sci. 2024;6(1):e172. 10.1002/ffo2.172.

[27] Niederberger M, Schifano J, Deckert S, Hirt J, Homberg A, Köberich S, et al. Delphi studies in social and health sciences-recommendations for an interdisciplinary standardized reporting (DELPHISTAR). Results of a Delphi study. PLoS ONE. 2024;19(8):e0304651. 10.1371/journal.pone.0304651. 39186713 10.1371/journal.pone.0304651 PMC11346927

[28] Clayton MJ. Delphi: a technique to harness expert opinion for critical decision-making tasks in education. Educ Psychol. 1997;17(4):373–86. 10.1080/0144341970170401.

[29] Haji FA, Khan R, Regehr G, Ng G, de Ribaupierre S, Dubrowski A. Operationalising elaboration theory for simulation instruction design: a Delphi study. Med Educ. 2015;49(6):576–88. 10.1111/medu.12726. 25989406 10.1111/medu.12726

[30] NHS Health Education England National Framework for Simulation-Based Education (SBE): NHS Health Education England. 2018:1–8. Available from: https://www.hee.nhs.uk/sites/default/files/documents/National%20framework%20for%20simulation%20based%20education.pdf.

[31] Cohen L, Manion L, Morrison K. Research methods in education. 8th ed. London & New York: Routledge. 2018.

[32] Knight J. International education hubs: Student, talent, knowledge-innovation models. Dordrecht: Springer Science & Business Media. 2013.

[33] Bovill C. Co-creation in learning and teaching: the case for a whole-class approach in higher education. Higher Educ. 2020;79(6):1023–37. 10.1007/s10734-019-00453-w.

[34] Zarandi N, Soares A, Alves H. Strategies, benefits and barriers– a systematic literature review of student co-creation in higher education. J Mark High Educ. 2024;34(2):895–919. 10.1080/08841241.2022.2134956.

[35] Humphrey-Murto S, Varpio L, Gonsalves C, Wood TJ. Using consensus group methods such as Delphi and nominal group in medical education research. Med Teach. 2017;39(1):14–9. 10.1080/0142159X.2017.1245856. 27841062 10.1080/0142159X.2017.1245856

[36] Qualtrics. June 2023 Provo, Utah, USA. 2023. https://www.qualtrics.com.

[37] Ringsted C, Hodges B, Scherpbier A. “The research compass”: an introduction to research in medical education: AMEE guide no. 56. Med Teach. 2011;33(9):695–709. 10.3109/0142159X.2011.595436. 21854147 10.3109/0142159X.2011.595436

[38] Barrios M, Guilera G, Nuño L, Gómez-Benito J. Consensus in the delphi method: what makes a decision change? Technol Forecast Soc Change. 2021;163:120484. 10.1016/j.techfore.2020.120484.

[39] Braun V, Clarke V. Using thematic analysis in psychology. Qual Res Psychol. 2006;3(2):77–101. 10.1191/1478088706qp063oa.

[40] Kiger ME, Varpio L. Thematic analysis of qualitative data: AMEE guide no. 131. Med Teach. 2020;42(8):846–54. 10.1080/0142159X.2020.1755030. 32356468 10.1080/0142159X.2020.1755030

[41] Nowell LS, Norris JM, White DE, Moules NJ. Thematic analysis: striving to meet the trustworthiness criteria. Int J Qual Methods. 2017;16(1):1609406917733847. 10.1177/1609406917733847.

[42] Braun V, Clarke V, Hayfield N, Terry G. Thematic Analysis. In: Liamputtong P, editor. Handbook of research methods in health social sciences. Singapore: Springer Singapore; 2019. p. 843–60.

[43] Braun V, Clarke V. Toward good practice in thematic analysis: Avoiding common problems and be(com)ing aknowingresearcher. Int J Transgender Health. 2022;24(1):1–6. 10.1080/26895269.2022.2129597. 10.1080/26895269.2022.2129597 PMC9879167 36713144

[44] Bastea A, Catalano H, Dohotaru A. An overview of distributed leadership and its shortcomings in educational settings. Educatia 21. 2023:114–26. 10.24193/ed21.2023.25.12.

[45] Das N, Dixit M. Distributed leadership models enhancing collaborative decision making in educational institutions. Stallion J Multidiscip Assoc Res Stud. 2024;3(1):25–31. 10.55544/sjmars.3.1.3.

[46] Motola I, Devine LA, Chung HS, Sullivan JE, Issenberg SB. Simulation in healthcare education: a best evidence practical guide. AMEE Guide No. 82. Med Teach. 2013;35(10):e1511–30. 10.3109/0142159X.2013.818632. 10.3109/0142159X.2013.818632 23941678

[47] Pottle J. Virtual reality and the transformation of medical education. Future Healthc J. 2019;6(3):181–5. 10.7861/fhj.2019-0036. 31660522 10.7861/fhj.2019-0036 PMC6798020

[48] Holmes L, Cresswell K, Williams S, Parsons S, Keane A, Wilson C, et al. Innovating public engagement and patient involvement through strategic collaboration and practice. Res Involv Engagem. 2019;5:30. 10.1186/s40900-019-0160-4. 31646001 10.1186/s40900-019-0160-4 PMC6802177

[49] Hasson F, Keeney S. Enhancing rigour in the Delphi technique research. Technol Forecast Soc Change. 2011;78(9):1695–704. 10.1016/j.techfore.2011.04.005.

[50] Chan KS, Zary N. Applications and challenges of implementing artificial intelligence in medical education: integrative review. JMIR Med Educ. 2019;5(1):e13930. 10.2196/13930. 31199295 10.2196/13930 PMC6598417

[51] Keeney S, McKenna HA, Hasson F. The Delphi technique in nursing and health research. Oxford: John Wiley & Sons. 2011.

[52] Hasson F, Keeney S, McKenna H. Research guidelines for the Delphi survey technique. J Adv Nurs. 2000;32(4):1008–15. 11095242

